# 4-Nitro-*N*-(8-quinol­yl)benzamide

**DOI:** 10.1107/S1600536808038038

**Published:** 2008-11-20

**Authors:** Gang Lei, Lin-Hai Jing, Li Zhou

**Affiliations:** a School of Chemistry and Chemical Engineering, China West Normal University, Nanchong 637002, People’s Republic of China

## Abstract

In the title compound, C_16_H_11_N_3_O_3_, the amide group is twisted away from the plane of the quinoline benzene ring by 3.93 (5)°, but is twisted away from the nitro­benzene ring by 22.68 (4)°. A weak intra­molecular C—H⋯O hydrogen bond is observed. In the crystal structure, mol­ecules are linked into a chain along the *a* axis by inter­molecular C—H⋯O hydrogen bonds.

## Related literature

For general background, see: Daoud *et al.* (2000 ▶); Westaway *et al.* (2006 ▶). For related structures, see: Lei *et al.* (2008*a* ▶,*b* ▶).
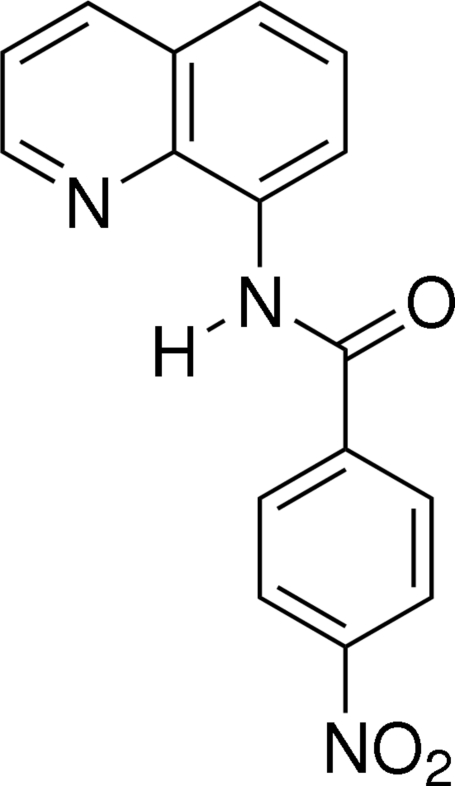


## Experimental

### 

#### Crystal data

C_16_H_11_N_3_O_3_
*M* *_r_* = 293.28
Monoclinic, 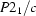

*a* = 7.5230 (15) Å
*b* = 25.031 (5) Å
*c* = 6.9596 (15) Å
β = 100.081 (3)°
*V* = 1290.3 (5) Å^3^
*Z* = 4
Mo *K*α radiation
μ = 0.11 mm^−1^
*T* = 93 (2) K
0.43 × 0.30 × 0.15 mm

#### Data collection

Rigaku R-AXIS RAPID diffractometer
Absorption correction: none
10531 measured reflections
2905 independent reflections
2542 reflections with *I* > 2σ(*I*)
*R*_int_ = 0.030

#### Refinement

*R*[*F* ^2^ > 2σ(*F* ^2^)] = 0.046
*wR*(*F* ^2^) = 0.135
*S* = 1.00
2905 reflections
199 parameters
H-atom parameters constrained
Δρ_max_ = 0.27 e Å^−3^
Δρ_min_ = −0.27 e Å^−3^

### 

Data collection: *RAPID-AUTO* (Rigaku, 2004 ▶); cell refinement: *RAPID-AUTO*; data reduction: *RAPID-AUTO*; program(s) used to solve structure: *SHELXS97* (Sheldrick, 2008 ▶); program(s) used to refine structure: *SHELXL97* (Sheldrick, 2008 ▶); molecular graphics: *XP* in *SHELXTL* (Sheldrick, 2008 ▶); software used to prepare material for publication: *SHELXL97*.

## Supplementary Material

Crystal structure: contains datablocks global, I. DOI: 10.1107/S1600536808038038/ci2725sup1.cif

Structure factors: contains datablocks I. DOI: 10.1107/S1600536808038038/ci2725Isup2.hkl

Additional supplementary materials:  crystallographic information; 3D view; checkCIF report

## Figures and Tables

**Table 1:** Hydrogen-bond geometry (Å, °)

*D*—H⋯*A*	*D*—H	H⋯*A*	*D*⋯*A*	*D*—H⋯*A*
C7—H7⋯O1	0.95	2.26	2.8672 (19)	121
C14—H14⋯O1^i^	0.95	2.34	3.2483 (18)	160
C17—H17⋯O3^ii^	0.95	2.38	3.3050 (18)	163

Symmetry codes: (i) 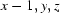; (ii) 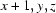.

**Fig. 1.**
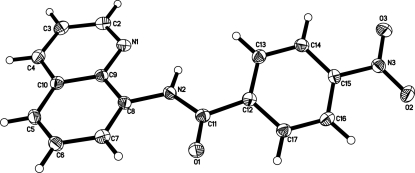
The molecular structure of the title compound, showing 50% probability displacement ellipsoids and the atomic numbering.

## supplementary crystallographic information

### Comment

Quinoline derivatives are a class of important compound as antagonists of TRPV1 (Westaway *et al.*, 2006) and a sensitive and specific probe for studying MRP-drug interactions (Daoud *et al.*, 2000). Previously, we have reported the crystal structures of (2-nitrophenyl)-*N*-(8-quinolyl)carboxamide (Lei *et al.*, 2008a) and (3-nitrophenyl)-*N*-(8-quinolyl)carboxamide (Lei *et al.*, 2008b). Now, we report here the crystal structure of the title compound (Fig.1).

Bond lengths and angles are normal. The quinoline ring system is planar, with a maximum deviation of 0.010 (7) Å for atom C5. As observed in related structures (Lei *et al.*, 2008a,b), the amide group is twisted away from the planes of the quinoline benzene ring and 4-nitro substituted benzene ring. The C5—C10 and C12—C17 planes form dihedral angles of 3.93 (5)° and 22.68 (4)°, respectively, with the O1/N2/C8/C11 plane. The C12—C17 and O2/O3/N3/C15 planes are inclined at an angle of 2.58 (7)°. The dihedral angle between the N1/C2-C10 and C12-C17 planes is 26.36 (3)°. An intramolecular C7—H7···O1 hydrogen bond is observed.

The crystal packing is stabilized by C—H···O hydrogen bonds (Table 1). These hydrogen bonds link the molecules into a chain along the *a* axis.

### Experimental

*p*-Nitrobenzoic acid (2 mmol) and an excess of thionyl chloride (3 mmol) in dioxane (20 ml) were boiled under reflux for 6 h. The solution was distilled under reduced pressure and a yellow solid was obtained. 8-Aminoquinoline (2 mmol) in tetrahydrofuran (20 ml) was added to the yellow solid and boiled under reflux for 6 h. The solution was then cooled to ambient temperature and filtered to remove the tetrahydrofuran. The precipitate was dissolved in dimethyl sulfoxide and allowed to stand for one month at ambient temperature, after which time white single crystals of the title compound suitable for X-ray diffraction were obtained.

### Refinement

All H atoms were placed in calculated positions, with C-H = 0.95 Å and N-H = 0.88 Å, and refined using a riding model, with *U*_iso_(H) = 1.2*U*_eq_(C,N).

### Crystal data

**Table:**

C_16_H_11_N_3_O_3_	*F*_000_ = 608
*M**_r_* = 293.28	*D*_x_ = 1.510 Mg m^−^^3^
Monoclinic, *P*2_1_/*c*	Mo *K*α radiation λ = 0.71073 Å
Hall symbol: -P 2ybc	Cell parameters from 3970 reflections
*a* = 7.5230 (15) Å	θ = 3.1–27.5º
*b* = 25.031 (5) Å	µ = 0.11 mm^−^^1^
*c* = 6.9596 (15) Å	*T* = 93 (2) K
β = 100.081 (3)º	Block, white
*V* = 1290.3 (5) Å^3^	0.43 × 0.30 × 0.15 mm
*Z* = 4	

### Data collection

**Table:**

Rigaku R-AXIS RAPID diffractometer	2542 reflections with *I* > 2σ(*I*)
Radiation source: fine-focus sealed tube	*R*_int_ = 0.030
Monochromator: graphite	θ_max_ = 27.5º
*T* = 93(2) K	θ_min_ = 3.1º
ω scans	*h* = −9→9
Absorption correction: none	*k* = −32→21
10531 measured reflections	*l* = −9→9
2905 independent reflections	

### Refinement

**Table:**

Refinement on *F*^2^	Secondary atom site location: difference Fourier map
Least-squares matrix: full	Hydrogen site location: inferred from neighbouring sites
*R*[*F*^2^ > 2σ(*F*^2^)] = 0.046	H-atom parameters constrained
*wR*(*F*^2^) = 0.135	*w* = 1/[σ^2^(*F*_o_^2^) + (0.0868*P*)^2^ + 0.086*P*] where *P* = (*F*_o_^2^ + 2*F*_c_^2^)/3
*S* = 1.00	(Δ/σ)_max_ = 0.001
2905 reflections	Δρ_max_ = 0.27 e Å^−^^3^
199 parameters	Δρ_min_ = −0.27 e Å^−^^3^
Primary atom site location: structure-invariant direct methods	Extinction correction: none

### Special details

**Table:**

Geometry. All e.s.d.'s (except the e.s.d. in the dihedral angle between two l.s. planes) are estimated using the full covariance matrix. The cell e.s.d.'s are taken into account individually in the estimation of e.s.d.'s in distances, angles and torsion angles; correlations between e.s.d.'s in cell parameters are only used when they are defined by crystal symmetry. An approximate (isotropic) treatment of cell e.s.d.'s is used for estimating e.s.d.'s involving l.s. planes.
Refinement. Refinement of *F*^2^ against ALL reflections. The weighted *R*-factor *wR* and goodness of fit *S* are based on *F*^2^, conventional *R*-factors *R* are based on *F*, with *F* set to zero for negative *F*^2^. The threshold expression of *F*^2^ > σ(*F*^2^) is used only for calculating *R*-factors(gt) *etc*. and is not relevant to the choice of reflections for refinement. *R*-factors based on *F*^2^ are statistically about twice as large as those based on *F*, and *R*- factors based on ALL data will be even larger.

### Fractional atomic coordinates and isotropic or equivalent isotropic displacement parameters (Å^2^)

**Table:**

	*x*	*y*	*z*	*U*_iso_*/*U*_eq_	
O1	0.69786 (14)	0.38934 (4)	0.50464 (16)	0.0233 (3)	
O2	−0.00273 (15)	0.55640 (4)	0.15111 (17)	0.0268 (3)	
O3	−0.19660 (14)	0.49876 (4)	0.22148 (16)	0.0286 (3)	
N1	0.38108 (16)	0.21834 (5)	0.42312 (18)	0.0192 (3)	
N2	0.51347 (16)	0.31652 (5)	0.46574 (18)	0.0188 (3)	
H2N	0.3993	0.3069	0.4397	0.023*	
N3	−0.04073 (16)	0.51288 (5)	0.21646 (18)	0.0202 (3)	
C2	0.3131 (2)	0.16969 (6)	0.3996 (2)	0.0220 (3)	
H2	0.1860	0.1663	0.3619	0.026*	
C3	0.4163 (2)	0.12257 (6)	0.4267 (2)	0.0225 (3)	
H3	0.3600	0.0886	0.4073	0.027*	
C4	0.5990 (2)	0.12671 (6)	0.4816 (2)	0.0212 (3)	
H4	0.6713	0.0954	0.5017	0.025*	
C5	0.8683 (2)	0.18552 (6)	0.5631 (2)	0.0208 (3)	
H5	0.9473	0.1556	0.5820	0.025*	
C6	0.9366 (2)	0.23613 (6)	0.5891 (2)	0.0212 (3)	
H6	1.0631	0.2410	0.6273	0.025*	
C7	0.8229 (2)	0.28130 (6)	0.5603 (2)	0.0199 (3)	
H7	0.8729	0.3161	0.5803	0.024*	
C8	0.64030 (19)	0.27499 (5)	0.5036 (2)	0.0170 (3)	
C9	0.56440 (19)	0.22250 (5)	0.4778 (2)	0.0172 (3)	
C10	0.6807 (2)	0.17763 (6)	0.5084 (2)	0.0187 (3)	
C11	0.54650 (19)	0.36987 (6)	0.4646 (2)	0.0183 (3)	
C12	0.38470 (19)	0.40539 (5)	0.4061 (2)	0.0176 (3)	
C13	0.2074 (2)	0.39106 (6)	0.4189 (2)	0.0192 (3)	
H13	0.1833	0.3569	0.4679	0.023*	
C14	0.06669 (19)	0.42655 (6)	0.3603 (2)	0.0196 (3)	
H14	−0.0540	0.4174	0.3698	0.023*	
C15	0.10717 (18)	0.47577 (6)	0.2876 (2)	0.0177 (3)	
C16	0.2814 (2)	0.49139 (6)	0.2769 (2)	0.0197 (3)	
H16	0.3051	0.5258	0.2295	0.024*	
C17	0.4202 (2)	0.45575 (6)	0.3369 (2)	0.0194 (3)	
H17	0.5410	0.4657	0.3308	0.023*	

### Atomic displacement parameters (Å^2^)

**Table:**

	*U*^11^	*U*^22^	*U*^33^	*U*^12^	*U*^13^	*U*^23^
O1	0.0184 (5)	0.0193 (5)	0.0318 (6)	−0.0021 (4)	0.0033 (5)	0.0004 (4)
O2	0.0276 (6)	0.0197 (6)	0.0326 (6)	0.0013 (4)	0.0041 (5)	0.0062 (5)
O3	0.0170 (6)	0.0307 (6)	0.0390 (7)	0.0022 (4)	0.0079 (5)	0.0071 (5)
N1	0.0178 (6)	0.0208 (6)	0.0201 (6)	−0.0022 (5)	0.0061 (5)	−0.0010 (5)
N2	0.0153 (6)	0.0159 (6)	0.0255 (7)	0.0000 (4)	0.0045 (5)	−0.0006 (5)
N3	0.0199 (6)	0.0209 (6)	0.0202 (6)	0.0015 (5)	0.0049 (5)	−0.0005 (5)
C2	0.0219 (8)	0.0245 (8)	0.0201 (8)	−0.0037 (6)	0.0055 (6)	−0.0010 (6)
C3	0.0285 (8)	0.0194 (7)	0.0201 (7)	−0.0043 (6)	0.0055 (6)	−0.0016 (6)
C4	0.0275 (8)	0.0183 (7)	0.0190 (7)	0.0024 (6)	0.0073 (6)	0.0004 (6)
C5	0.0205 (7)	0.0221 (7)	0.0198 (7)	0.0055 (6)	0.0037 (6)	0.0022 (6)
C6	0.0181 (7)	0.0252 (8)	0.0207 (7)	0.0023 (6)	0.0044 (6)	0.0013 (6)
C7	0.0213 (7)	0.0194 (7)	0.0198 (7)	−0.0020 (6)	0.0053 (6)	−0.0009 (6)
C8	0.0180 (7)	0.0183 (7)	0.0157 (7)	0.0022 (5)	0.0056 (6)	0.0001 (5)
C9	0.0179 (7)	0.0195 (7)	0.0150 (7)	0.0011 (5)	0.0051 (6)	−0.0004 (5)
C10	0.0224 (7)	0.0201 (7)	0.0144 (7)	0.0011 (6)	0.0054 (6)	0.0007 (5)
C11	0.0194 (7)	0.0186 (7)	0.0173 (7)	−0.0009 (6)	0.0047 (6)	−0.0011 (5)
C12	0.0180 (7)	0.0174 (7)	0.0173 (7)	−0.0012 (5)	0.0033 (6)	−0.0022 (5)
C13	0.0215 (7)	0.0173 (7)	0.0192 (7)	−0.0011 (6)	0.0041 (6)	−0.0005 (6)
C14	0.0183 (7)	0.0197 (7)	0.0214 (7)	−0.0013 (6)	0.0055 (6)	−0.0013 (6)
C15	0.0183 (7)	0.0174 (7)	0.0171 (7)	0.0025 (5)	0.0022 (6)	−0.0010 (5)
C16	0.0224 (8)	0.0173 (7)	0.0196 (7)	−0.0032 (6)	0.0046 (6)	0.0004 (6)
C17	0.0190 (7)	0.0188 (7)	0.0210 (7)	−0.0024 (6)	0.0047 (6)	−0.0012 (6)

### Geometric parameters (Å, °)

**Table:**

O1—C11	1.2250 (17)	C6—C7	1.411 (2)
O2—N3	1.2332 (15)	C6—H6	0.95
O3—N3	1.2311 (16)	C7—C8	1.370 (2)
N1—C2	1.3198 (18)	C7—H7	0.95
N1—C9	1.3689 (18)	C8—C9	1.4311 (19)
N2—C11	1.3587 (18)	C9—C10	1.4166 (19)
N2—C8	1.4045 (17)	C11—C12	1.505 (2)
N2—H2N	0.88	C12—C17	1.3921 (19)
N3—C15	1.4673 (18)	C12—C13	1.399 (2)
C2—C3	1.406 (2)	C13—C14	1.388 (2)
C2—H2	0.95	C13—H13	0.95
C3—C4	1.365 (2)	C14—C15	1.386 (2)
C3—H3	0.95	C14—H14	0.95
C4—C10	1.413 (2)	C15—C16	1.382 (2)
C4—H4	0.95	C16—C17	1.381 (2)
C5—C6	1.367 (2)	C16—H16	0.95
C5—C10	1.410 (2)	C17—H17	0.95
C5—H5	0.95		
			
C2—N1—C9	117.02 (12)	N1—C9—C10	123.15 (13)
C11—N2—C8	127.57 (12)	N1—C9—C8	117.72 (12)
C11—N2—H2N	116.2	C10—C9—C8	119.12 (13)
C8—N2—H2N	116.2	C5—C10—C4	123.60 (13)
O3—N3—O2	123.20 (12)	C5—C10—C9	119.48 (13)
O3—N3—C15	118.58 (12)	C4—C10—C9	116.93 (14)
O2—N3—C15	118.20 (12)	O1—C11—N2	123.60 (14)
N1—C2—C3	124.36 (14)	O1—C11—C12	120.14 (13)
N1—C2—H2	117.8	N2—C11—C12	116.24 (12)
C3—C2—H2	117.8	C17—C12—C13	119.82 (13)
C4—C3—C2	118.62 (14)	C17—C12—C11	115.68 (13)
C4—C3—H3	120.7	C13—C12—C11	124.50 (13)
C2—C3—H3	120.7	C14—C13—C12	120.28 (13)
C3—C4—C10	119.91 (14)	C14—C13—H13	119.9
C3—C4—H4	120.0	C12—C13—H13	119.9
C10—C4—H4	120.0	C15—C14—C13	118.12 (13)
C6—C5—C10	120.06 (13)	C15—C14—H14	120.9
C6—C5—H5	120.0	C13—C14—H14	120.9
C10—C5—H5	120.0	C16—C15—C14	122.82 (13)
C5—C6—C7	121.27 (14)	C16—C15—N3	118.23 (13)
C5—C6—H6	119.4	C14—C15—N3	118.94 (12)
C7—C6—H6	119.4	C17—C16—C15	118.37 (13)
C8—C7—C6	120.06 (13)	C17—C16—H16	120.8
C8—C7—H7	120.0	C15—C16—H16	120.8
C6—C7—H7	120.0	C16—C17—C12	120.56 (13)
C7—C8—N2	125.63 (13)	C16—C17—H17	119.7
C7—C8—C9	119.99 (12)	C12—C17—H17	119.7
N2—C8—C9	114.38 (12)		
			
C9—N1—C2—C3	−0.1 (2)	C8—C9—C10—C4	179.81 (12)
N1—C2—C3—C4	0.2 (2)	C8—N2—C11—O1	−2.6 (2)
C2—C3—C4—C10	−0.5 (2)	C8—N2—C11—C12	176.13 (12)
C10—C5—C6—C7	−0.6 (2)	O1—C11—C12—C17	21.26 (19)
C5—C6—C7—C8	−0.6 (2)	N2—C11—C12—C17	−157.50 (13)
C6—C7—C8—N2	−178.40 (13)	O1—C11—C12—C13	−158.21 (14)
C6—C7—C8—C9	1.4 (2)	N2—C11—C12—C13	23.0 (2)
C11—N2—C8—C7	4.8 (2)	C17—C12—C13—C14	0.9 (2)
C11—N2—C8—C9	−174.98 (13)	C11—C12—C13—C14	−179.67 (13)
C2—N1—C9—C10	0.3 (2)	C12—C13—C14—C15	0.7 (2)
C2—N1—C9—C8	179.93 (12)	C13—C14—C15—C16	−2.1 (2)
C7—C8—C9—N1	179.29 (12)	C13—C14—C15—N3	177.21 (12)
N2—C8—C9—N1	−0.91 (19)	O3—N3—C15—C16	178.40 (13)
C7—C8—C9—C10	−1.0 (2)	O2—N3—C15—C16	−0.28 (19)
N2—C8—C9—C10	178.77 (12)	O3—N3—C15—C14	−0.90 (19)
C6—C5—C10—C4	−178.98 (13)	O2—N3—C15—C14	−179.58 (13)
C6—C5—C10—C9	1.0 (2)	C14—C15—C16—C17	1.7 (2)
C3—C4—C10—C5	−179.42 (13)	N3—C15—C16—C17	−177.59 (12)
C3—C4—C10—C9	0.6 (2)	C15—C16—C17—C12	0.0 (2)
N1—C9—C10—C5	179.52 (12)	C13—C12—C17—C16	−1.3 (2)
C8—C9—C10—C5	−0.1 (2)	C11—C12—C17—C16	179.23 (12)
N1—C9—C10—C4	−0.5 (2)		

### Hydrogen-bond geometry (Å, °)

**Table:**

*D*—H···*A*	*D*—H	H···*A*	*D*···*A*	*D*—H···*A*
C7—H7···O1	0.95	2.26	2.8672 (19)	121
C14—H14···O1^i^	0.95	2.34	3.2483 (18)	160
C17—H17···O3^ii^	0.95	2.38	3.3050 (18)	163

Symmetry codes: (i) *x*−1, *y*, *z*; (ii) *x*+1, *y*, *z*.
